# CRISPR/Cas12a Based Rapid Molecular Detection of Acute Hepatopancreatic Necrosis Disease in Shrimp

**DOI:** 10.3389/fvets.2021.819681

**Published:** 2022-01-25

**Authors:** Chenglong Li, Nan Lin, Zhihua Feng, Minhua Lin, Biyun Guan, Kunsen Chen, Wangwang Liang, Qiaohuang Wang, Miaomiao Li, Yu You, Qi Chen

**Affiliations:** ^1^Fujian Key Laboratory of Innate Immune Biology, Biomedical Research Center of South China, College of Life Science, Fujian Normal University, Fuzhou, China; ^2^Fujian Provincial Fisheries Technology Extension Center, Fuzhou, China

**Keywords:** acute hepatopancreatic necrosis disease (AHPND), CRISPR/Cas12a, recombinase polymerase amplification (RPA), lateral flow strip (LFS), shrimp

## Abstract

Acute hepatopancreatic necrosis disease (AHPND), formerly called early mortality syndrome (EMS), causes high mortality in cultured penaeid shrimp, particularly *Penaeus vannamei* and *Penaeus monodon*. AHPND is mainly caused by *Vibrio* species carrying the pVA1 plasmid encoding the virulence genes Photorhabdus insect-related (*pir*) *pir*^*VP*^*A* and *pir*^*VP*^*B*. We developed a new molecular assay that combines recombinase polymerase amplification (RPA) and CRISPR/Cas12a technology (RPA-CRISPR/Cas12a) to detect *pir*^*VP*^*A* and *pir*^*VP*^*B*, with a fluorescent signal result. The fluorescence RPA-CRISPR/Cas12a assay had a detection limit of 20 copies/μL for *pir*^*VP*^*A* and *pir*^*VP*^*B*. To improve usability and visualize RPA-CRISPR/Cas12a assay results, a lateral flow strip readout was added. With the lateral flow strip, the RPA-CRISPR/Cas12a assay had a lower limit of detection of 200 copies/μL (0.3 fmol/L). The lateral flow assay can be completed in 2 h and showed no cross-reactivity with pathogens causing other shrimp diseases. In a field test of 60 shrimp samples, the RPA-CRISPR/Cas12a lateral flow assay showed 92.5% positive predictive agreement and 100% negative predictive agreement. As the new RPA-CRISPR/Cas12a assay is rapid, specific, and does not require complicated experimental equipment, it may have important field applications for detecting AHPND in farmed shrimp.

## Introduction

As the demand for shrimp continues to increase worldwide, Asia has become a dominant supplier of farmed penaeid shrimp, in particular *Penaeus vannamei* (*P. vannamei*) and *Penaeus monodon* (*P. monodon*). In 2018, the production of aquaculture in Asia totaled 73 million tons, accounting for 88.7% of the world's total production (1). In Southeast Asia alone, production was 5.37 million tons, with *P. vannamei* and *P. monodon* accounting for the majority of total penaeid production (1, 2). The primary threat to the Asian shrimp supply is disease caused by viruses, bacteria, and parasites (3), which have led to losses of nearly 80% in recent years. Acute hepatopancreatic necrosis disease (AHPND), formerly called early mortality syndrome (EMS), is caused by infection with *Vibrio* species and leads to high mortality rates in cultured penaeid shrimp. Shrimp with AHPND show slow growth, massive sloughing of hepatopancreatic tubule epithelial cells, and an empty stomach and midgut (4, 5). The first outbreak of AHPND in China occurred in 2009, and quickly spread to Vietnam, Malaysia, Thailand, and Mexico (4, 6, 7). In Asia, the economic loss associated with AHPND in cultured penaeid shrimp was ~US$44 billion in 2010–2016 (6). In Vietnam, the losses due to the outbreak peaked in 2015 at US$97.96 million; in Thailand, losses from 2012 to 2015 totaled US$5.01 billion, including 100,000 in lost jobs (6). With poor management and reliance on time-consuming detection methods, infections with *Vibrio* and other pathogens, such as the white spot syndrome virus (WSSV, a member of the Nimaviridae family) (8–10) and the parasite enterocytozoon hepatopenaei (EHP) (11), will continue to cause serious economic losses in the shrimp culture industry. Timely detection and prevention of AHPND spread in farmed shrimp is thus critical to mitigate the associated economic losses.

Many *Vibrio* species have been isolated from AHPND-infected shrimp, including *Vibrio parahaemolyticus* (12–14), *Vibrio harveyi* (15), *Vibrio owensii* (16), and *Vibrio campbellii* (17). These pathogens all carry an AHPND-related plasmid encoding the Photorhabdus insect-related (*pir*) *pir*^*VP*^*A* and *pir*^*VP*^*B* genes (GenBank Accession No. KM067908.1) (18). Several groups have pursued molecular testing to detect AHPND by targeting *pir*^*VP*^*A* and *pir*^*VP*^*B*, using methods including polymerase chain reaction (PCR), nested PCR, loop-mediated isothermal amplification (LAMP), recombinase polymerase amplification (RPA), and real-time quantitative PCR (qPCR) (19–22). The limit of detection (LOD) of PCR is 10 pg and nested PCR is 100 fg. With their high sensitivity and specificity, nested PCR and qPCR have been accepted by many agencies as the gold standard tests to diagnose AHPND (20, 23). Unlike PCR and nested PCR, isothermal amplification methods such as LAMP and RPA amplify nucleotides at a constant temperature and thus do not require thermocyclers. The RPA reaction comprises a recombinase, a single-stranded DNA-binding protein (SSB), a strand-displacing polymerase, two primers, and a constant temperature of 37–42°C (24). The LAMP reaction needs 4–6 primers and is run at 65°C to amplify target DNA (25). Isothermal amplification methods are rapid and sensitive and are often combined lateral flow strip technology to simplify its use in field-based assays, with test results read directly by the user (26). LAMP may produce non-specific amplicons that lead to false positive test results, and maintaining the test temperature at 65°C can be difficult. Therefore, RPA may be the better choice for developing rapid, sensitive, and simple molecular tests for field use (27).

In recent years, the CRISPR (Clustered Regularly Interspaced Short Palindromic Repeats)/Cas9 (CRISPR-associated protein) system has emerged as a powerful tool for gene editing. In the type II CRISPR/Cas system, a guide crRNA (CRISPR RNA) binds with Cas9 to form a Cas9-crRNA complex that then binds to target DNA. Cas9 recognizes a protospacer adjacent motif (PAM) sequence near the target DNA sequence, and activated Cas9 cleaves the target DNA to produce a double strand break (DSB) (28–30). Based on this, Cas9 has been applied to genome-scale screening (31) and deactivated Cas9 (dCas9) with gene activation/repression elements has been used to regulate gene transcription (32). Cas12 and Cas13 have an extra enzymatic activity called collateral cleavage that has implications for molecular assay development. Activated Cas12 possesses single-stranded DNA (ssDNA) cleavage activity (33), while Cas13 possesses single-stranded RNA (ssRNA) cleavage activity (34). ssDNA with a linked fluorophore and quencher at either end (called an FQ reporter, FAM-TTATT-TAMRA) can be cleaved by Cas12 in the presence of target DNA (35). Alternatively, the labeled reporter can be linked with biotin (called an FB reporter, FAM-TTATT-Biotin), which can been detected using lateral flow test strip. The technologies based on Cas12 and Cas13 collateral cleavage activity have been used to detect nCOV-2019, African swine fever virus (ASFV), Zika virus (ZIKV), and Dengue virus (DENV) (36–38).

In this study, we developed a novel molecular assay that combines the CRISPR/Cas12a system with RPA and lateral flow technology to detect AHPND virulence genes in cultured penaeid shrimp (*P. vannamei* and *P. monodon*). We found that a CRISPR/Cas12a based assay alone did not achieve a sufficiently low LOD for the *pir*^*VP*^*A* and *pir*^*VP*^*B* target genes. Thus, we added RPA to pre-amplify the target genes. Furthermore, to visualize the assay results, we combined our RPA-CRISPR/Cas12a assay with lateral flow strip (LFS) technology. Our new molecular assay (LFS-based RPA-CRISPR/Cas12a) has the potential to detect AHPND with high accuracy without the need for lab equipment, providing a convenient and simple method that can be deployed to the field for rapid AHPND detection in cultured shrimp.

## Materials and Methods

### Primers and crRNAs Design

PCR and RPA primers targeting the AHPND virulence genes *pir*^*VP*^*A* and *pir*^*VP*^*B* were designed based on the published sequence of pVA1 plasmid (GenBank Accession No. KM067908.1) in the National Center for Biotechnology Information (NCBI) database.

Cas12a crRNA consists of a 21 bp spacer and a 20 bp direct repeat (DR). For the T-rich (5′-TTTN-3′) protospacer adjacent motif (PAM) restriction, four crRNAs targeting the genes *pir*^*VP*^*A* and *pir*^*VP*^*B* were designed and validated. All primers and probes were purchased and synthesized by Sangon Biotech (Shanghai, China). The primers and DNA oligos used in this study were listed in Supplementary Tables 1, 2.

### *Vibrio* Culture and Plasmid Construction

AHPND-causing *Vibrio* was gifted from Fujian Provincial Fisheries Technology Extension Center (Fujian, China). *Vibrio* was cultured in Tryptic Soy Broth (TSB), at 37°C, 180 rpm overnight. *Vibrio* total DNA was extracted using the TaKaRa MiniBEST Viral RNA/DNA Extraction Kit (TaKaRa, China) according to the manufacturer's protocol. PCR primers (Supplementary Table 1) PirAB-F1 and PirAB-R1 were used to amplify the virulence genes, with an amplicon size of 1,794 bp. This amplicon was cloned into pMD19-T vector (TaKaRa, China) to create the recombinant plasmid pUC19-PirAB. After transformation into *E. coli* DH5α, the recombinant plasmid was extracted using a kit (DP118-02) purchased from TIANGEN BIOTECH (Beijing, China). The plasmid was measured with NanoDrop 2000C (Thermo Fisher, United States) at A260/280 and stored at −20°C.

### Preparation of Cas12a crRNA

The DNA templates for *in vitro* T7 transcription were synthesized by Sangon (Shanghai, China), and listed in Supplementary Table 2. The DNA template was comprised of T7 promoter and crRNA. crRNA was transcribed following the manufacturer's protocol with a T7 *in vitro* transcription kit (VK010) purchased from VIEWSOLID (Beijing, China). After transcribing at 37°C for 6 h, the products were mixed with 75% ethanol and incubated at −20°C overnight. Following centrifugation at 13,000 rpm for 30 min, the supernatant was discarded and RNA products were dissolved in RNase-free ddH_2_O and stored at −80°C for subsequent tests.

### CRISPR/Cas12a *in vitro* Cleavage Assay

Cas12a cleavage assays were performed in a PCR tube in a total reaction volume of 20 μL. One microlitre Cas12a (20 μM) and 1 μL crRNA (10 μM) were pre-mixed and incubated at 37°C for 15 min. Cas buffer, 100 ng of PCR products (primers: PirAB-F1 and PirAB-R1, products: 1,794 bp), and RNase-free ddH_2_O were then added to the reaction tube and incubated at 37°C for an additional 15 min. To stop the reaction, 3 μL DNA Loading Buffer was added and the tube was heated to 65°C for 5 min. Reaction products were verified by 2% agarose gel electrophoresis.

### CRISPR/Cas12a Fluorescence Assay and Optimization

The Cas12a fluorescence assay was performed by mixing Cas12a, crRNA, FQ reporter, and Cas buffer in a 384-well plate and incubating at 37°C for 45 min. The fluorescence signal was detected by QuantStudio 6 Flex (Applied Biosystems, USA) in the FAM channel every 60 s, with three replicates run in each reaction. The reaction conditions were optimized based on the assay performance using various concentrations of Cas12a and crRNA.

### Recombinase Polymerase Amplification and Primers Optimization

RPA was performed in a 50 μL tube containing the forward primer, reverse primer, primer-free rehydration buffer, DNA template, and magnesium acetate and using the Twistamp™ Basic Kit following the manufacturer's instructions (Babraham, UK). RPA products were purified and verified by 2% agarose gel electrophoresis. Various pairs of RPA primers (Supplementary Table 1) were tested to optimize the assay.

### Sensitivity of RPA-CRISPR/Cas12a Assay and qPCR

The sensitivity of our new RPA-CRISPR/Cas12a assay was tested using a 10-fold serial dilution panel of plasmid pUC19-PirAB (from 2 copies/μL to 2 × 10^7^ copies/μL). RPA products were used as the DNA template in the subsequent CRISPR/Cas12a fluorescence assay.

The performance of the assay was also compared to real-time qPCR as the current gold standard for detection of AHPND. qPCR primers (Supplementary Table 1) were PirAB-qPCR-F and PirAB-qPCR-R. The reaction was performed using FastStart™ Universal SYBR® Green Master (ROX) purchased from Roche (Roche, China) following the manufacturer's instruction. The qPCR reaction and readout of fluorescence were run on the StepOne system (Applied Biosystems, USA) with following the cycle conditions: 95°C for 5 min; 40 cycles of denaturation at 95°C for 10 s; annealing and amplification at 60°C for 30 s.

### RPA-CRISPR/Cas12a Assay With Lateral Flow Strip Technology

For direct readout of the assay result, we coupled LFS technology with the new RPA-CRISPR/Cas12a assay to create the LFS-based RPA-CRISPR/Cas12a assay. The LFS-based assay used the same reagents as the fluorescence-based assay except for the replacement of the FQ reporter with an FB reporter. The reaction was performed in a 1.5 mL tube containing Cas12a and crRNA. After a 10-min incubation, the reaction was mixed with FB reporter, Cas buffer, and DNA template, and incubated at 37°C for 30 min. Then, a lateral flow strip was added to the tube and the result was read in 10 min. The control band is close to the sample pad, while the test band appears at the top of the strip, away from the sample pad. Appearance of a red test band indicated a positive result; any other result was considered negative.

### Specificity of the RPA-CRISPR/Cas12a Assay

Several shrimp pathogens including WSSV and EHP were used to test cross-reactivity in the new RPA-CRISPR/Cas12a assays (fluorescence and LFS-based). All pathogens were gifted from Fujian Provincial Fisheries Technology Extension Center (Fujian, China). After RPA amplification of target DNA, the RPA products were directly used in subsequent fluorescence and lateral flow assays.

### Detection of AHPND in Field Samples

To assess the field performance of the new LFS-based RPA-CRISPR/Cas12a assay to detect AHPND-causing *Vibrio* strains, 60 shrimp samples were tested and the results were compared to the real-time qPCR assay. Total DNA was extracted from hepatopancreas (HP) tissues of shrimp samples using the TaKaRa MiniBEST viral RNA/DNA extraction kit (TaKaRa, China) following the manufacturer's protocols. Briefly, 800 μL of PBS solution was added to the shrimp sample in a 2 mL lysing matrix tube, the tissue was disrupted for 40 s using an MP Biomedical Fast-Prep®-24, and 200 μL of lysate was used for DNA extraction.

### Statistical Analysis

Software GraphPad Prism 6.01 (GraphPad Software, San Diego, CA) was used for statistical analyses and graphing. Student *t*-tests were performed and data were presented as mean ± SD, with error bars representing the standard deviation of at least three independent experiments.

## Results

### Cas12a *in vitro* Cleavage

**Figure 1:**
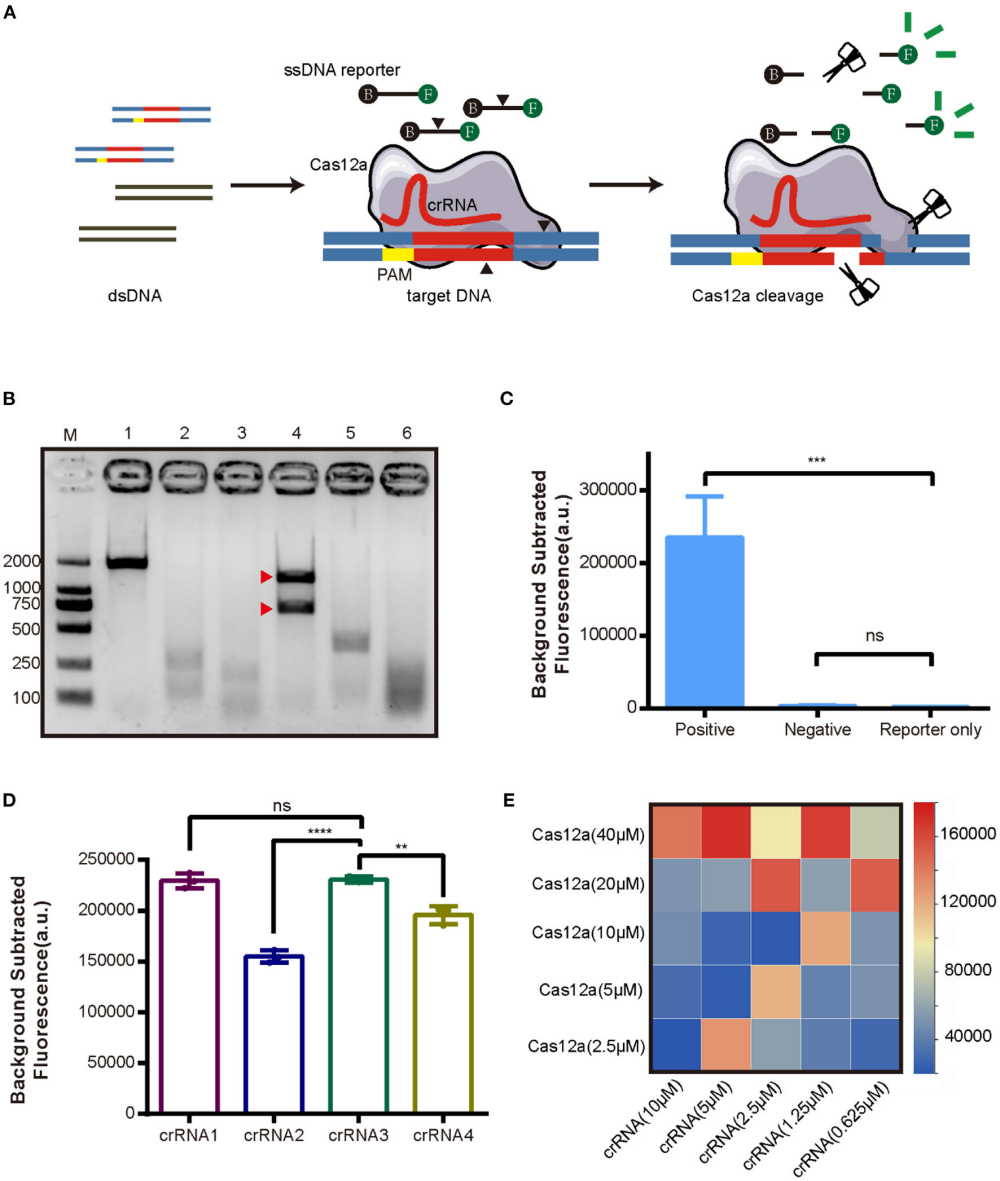
Development of the CRISPR/Cas12a assay for detection of AHPND virulence genes *Pir*^*VP*^*A* and *Pir*^*VP*^*B*. **(A)** Schematic diagram of the CRISPR/Cas12a. **(B)** Cleavage activity of crRNA-guided Cas12a. M: DL 2000 DNA marker; 1: intact dsDNA template (1794 bp); 2-5: The cleavage products of dsDNA template induced by crRNA1, crRNA2, crRNA3, crRNA4; 6: The cleavage products of dsDNA template induced by mixture of crRNA1 to crRNA4. The expected cleavage products are marked by red arrows. **(C)** Validation of collateral cleavage activity of Cas12a by fluorescence assay. The DNA template in positive group was plasmid pUC19-PirAB, and negative group was blank vector pUC19, while reporter only group was reaction without DNA template. **(D)** Comparison of different crRNAs targeting *Pir*^*VP*^*A* and *Pir*^*VP*^*B* by fluorescence assay. **(E)** Heatmap showing the optimal concentrations of Cas12a and crRNA based on fluorescence intensity. Each row presents the concentration of Cas12a used, each column presents the concentration of crRNA used. Error bars represent the standard deviation from three independent experiments. ^**^p < 0.01, ^***^p < 0.001, ^****^p < 0.0001 and *ns*, not significant.

As the first step in establishing the new CRISPR/Cas12a assay for AHPND (Figure 1), Cas12a cleavage activity was assessed in an *in vitro* cleavage assay. Cas12a protein was expressed in *E. coli* BL21 and purified with Ni-NTA resin (Supplementary Figure 2). Virulence genes *Pir*^*VP*^*A* and *Pir*^*VP*^*B* were cloned into pMD19-T vector (pUC19-PirAB) and verified *via* sequencing (Supplementary Figure 1). Four crRNAs were tested to target the virulence genes *Pir*^*VP*^*A* and *Pir*^*VP*^*B*. The results of the *in vitro* cleavage assay are shown in Figure 1B, intact dsDNA template (1,794 bp) was cleaved by Cas12a in the presence of crRNA, indicated that all four crRNAs enable Cas12a to cleave the target DNA template, crRNA3 induced a specific on-target cleavage with the expected cleave products of 1,142 and 652 bp, respectively, while the other crRNAs did not.

### CRISPR/Cas12a Reaction Optimization

We next assessed Cas12a collateral cleavage activity, specifically, its single-stranded DNase activity. A ssDNA labeled with fluorophore (FAM) and quencher (TAMRA) at each end, called an FQ reporter, was used to assess Cas12a collateral cleavage based on a fluorescent signal readout. The concentration of FQ reporter was 250 nM (Supplementary Figure 3). The plasmid pUC19-PirAB was used as a positive template, the blank vector pUC19 was used as a negative template, and no DNA template (reporter-only group) served as a negative control. The results (Figure 1C) showed that pUC19-PirAB produced a significant fluorescence signal (*p* < 0.001) whereas the blank vector pUC19 template showed no significant signal relative to the reporter-only group.

Because the four crRNAs target different sites on *Pir*^*VP*^*A* and *Pir*^*VP*^*B*, they may induce different levels of fluorescence intensity in the CRISPR/Cas12a assay. To optimize the assay, we compared the activity with each of the four crRNAs. Figure 1D indicates that crRNA1 and crRNA3 outperformed the other crRNAs in terms of fluorescence readout. Based upon the results of Cas12a *in vitro* cleavage (Figure 1B), thus, we selected crRNA3 for further assay development. We then optimized the concentration ratio of Cas12a to crRNA3; as shown in the heatmap (Figure 1E), a ratio of 40 μM Cas12a to 5 μM crRNA induced the highest fluorescence intensity.

### RPA-CRISPR/Cas12a Fluorescence Assay Development

**Figure 2:**
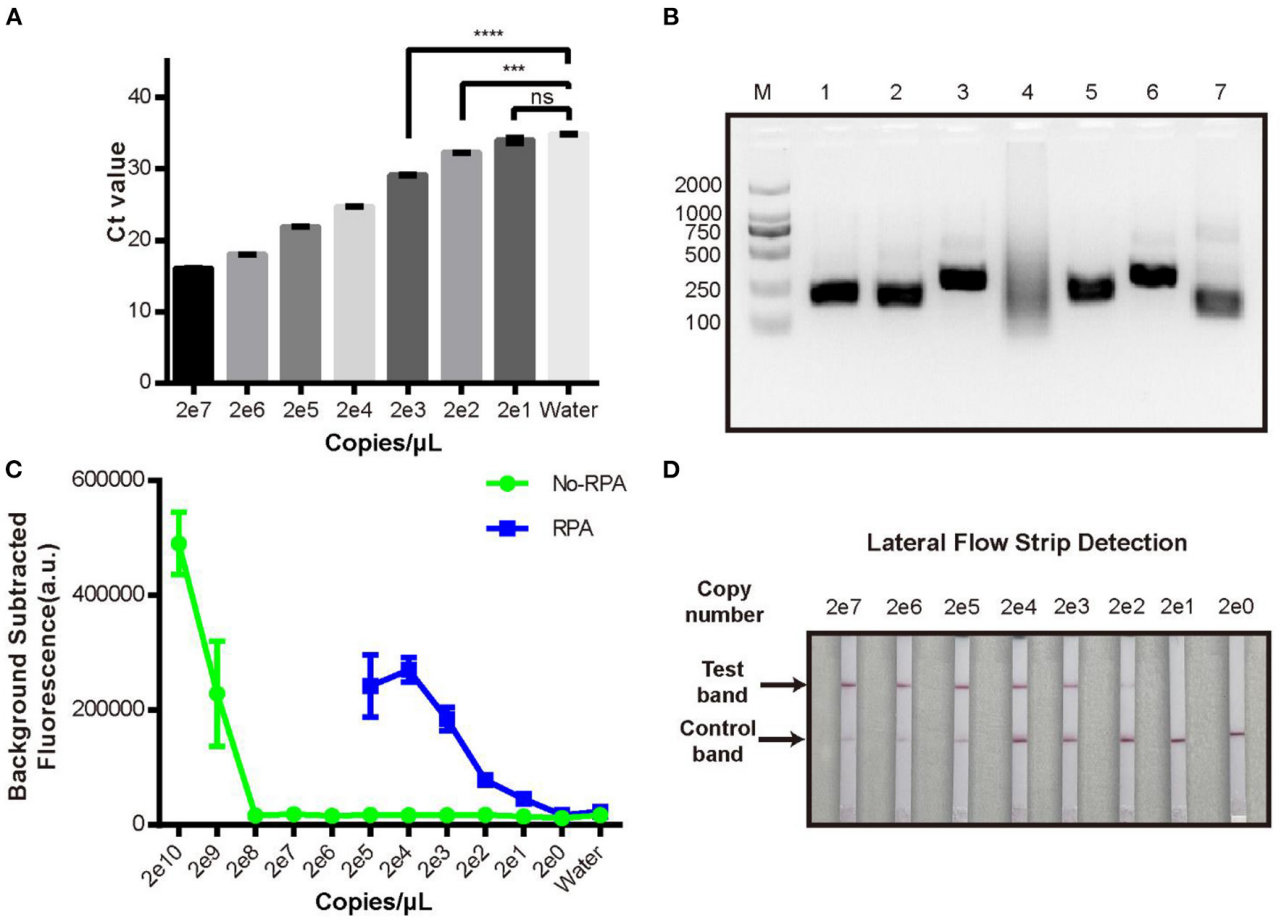
Limit of detection analysis of the new RPA-CRISPR/Cas12a fluorescence assay. **(A)** qPCR detection of pUC19-PirAB plasmid at various concentrations from 2 copies/μL to 2 × 10^7^ copies/μL. **(B)** Analysis of different RPA primers for pre-amplification of the target DNA. M: DL 2000 DNA marker; 1-6: the RPA product derived by using each of PirAB-RPA-F1/R1 to PirAB-RPA-F6/R6 primers; 7: RPA positive control. **(C)** Limit of detection of the pUC19-PirAB plasmid with or without RPA prior to the CRISPR/Cas12a fluorescence assay. **(D)** Limit of detection when lateral flow strip technology was combined with the RPA-CRISPR/Cas12a assay for detection of pUC19-PirAB plasmid from 2 copies to 2 × 10^7^ copies. Error bars represent the standard deviation from three independent experiments. ^***^p < 0.001, ^****^p < 0.0001 and *ns*, not significant.

We then evaluated the sensitivity of the new CRISPR/Cas12a assay compared with real-time qPCR to detect a dilution series of plasmid pUC19-PirAB. The limit of detection (LOD) of real-time qPCR was 200 copies/μL (Figure 2A) and of the CRISPR/Cas12a assay was 2 × 10^9^ copies/μL (Figure 2C). Thus, the CRISPR/Cas12a assay alone would be unable to detect *Vibrio* gene targets when present in low copy numbers.

To improve the LOD of the CRISPR/Cas12a assay, we added a recombinase polymerase amplification (RPA) step to pre-amplify the target DNA. Six RPA primers (Supplementary Table 1) were tested and run on 2% agarose gel (Figure 2B). Except for the PirAB-RPA-F4/PirAB-RPA-R4 primer set, all other RPA primers successfully amplified the target DNA. The PirAB-RPA-F3/PirAB-RPA-R3 primer set was chosen for further assay development since this pair of primer can induce more specific amplification products.

The LOD of the CRISPR/Cas12a assay was compared with and without RPA pre-amplification using the same serial dilution panel of plasmid pUC19-PirAB. Two microliter of RPA product was used as the template for RPA-CRISPR/Cas12a assay. The LOD of the RPA-CRISPR/Cas12a assay reached 20 copies/μL, lower than that achieved with real-time qPCR (Figure 2C).

**Figure 3:**
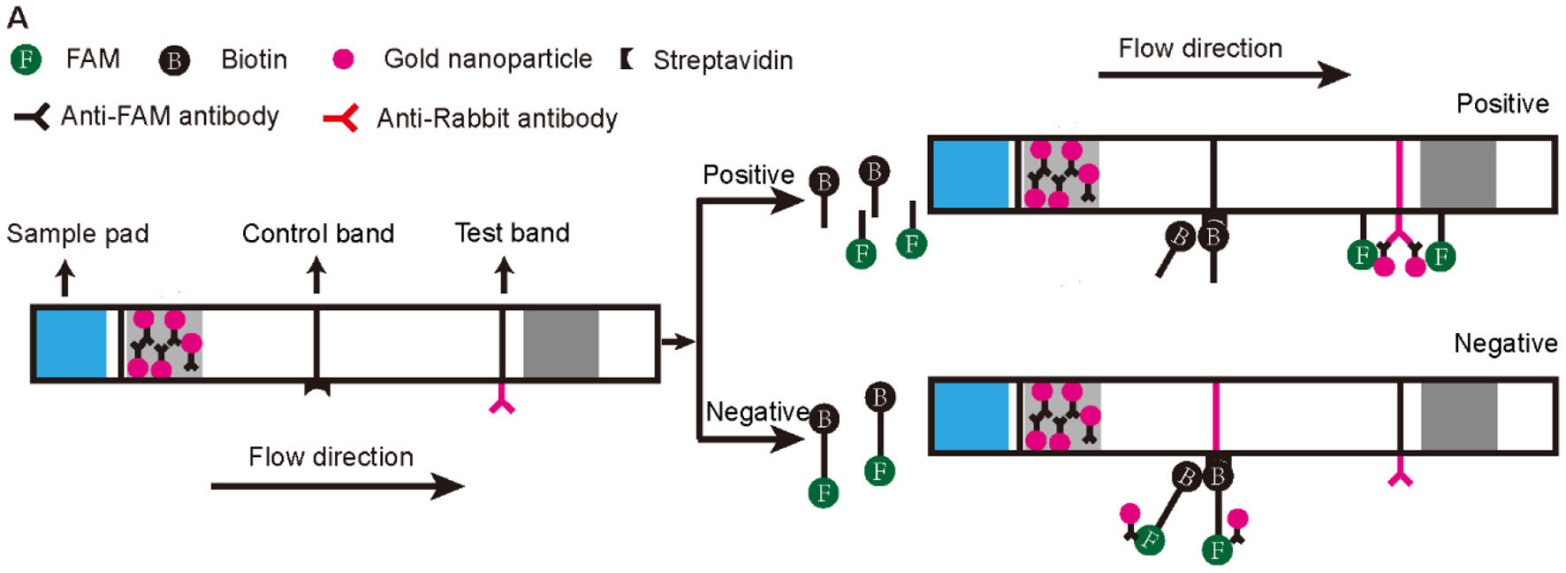
Schematic diagram of the lateral flow test strip technology applied to the RPA-CRISPR/Cas12a assay. The first line on the strip (Control band) is fixed with streptavidin, which can bind biotin. The second line on the strip (Test band) is fixed with Anti-Rabbit antibody that binds Anti-FAM antibody. Appearance of a red color test band indicates a positive result. Any other result is considered negative.

To make the RPA-CRISPR/Cas12a assay useful for rapid detection of AHPND in field-based settings, we combined it with lateral flow strip (LFS) technology to create the LFS-based RPA-CRISPR/Cas12a assay (Figure 3). We switched the FQ reporter with an FB reporter as the target for Cas12a collateral cleavage. Using the pUC19-PirAB plasmid dilution panel, the LFS-based RPA-CRISPR/Cas12a assay had an LOD of 200 copies/μL, comparable to real-time qPCR (Figure 2D).

### Field Performance of the New RPA-CRISPR/Cas12a Assay for AHPND

**Figure 4:**
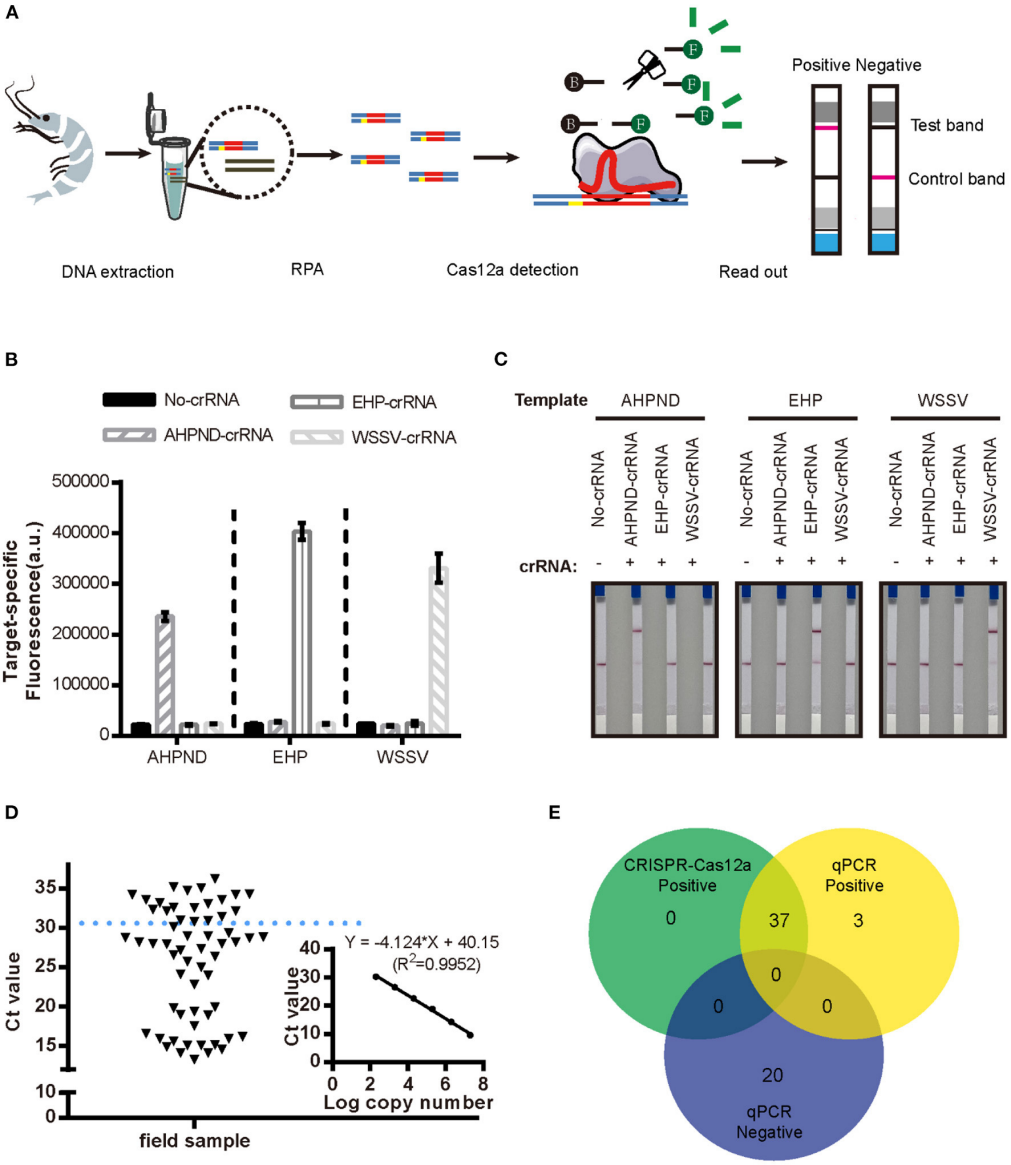
Detection of AHPND in field samples using the new LFS-based RPA-CRISPR/Cas12a assay compared to real-time qPCR. **(A)** Schematic diagram of the assay process using field samples. Specificity of the **(B)** fluorescence-based and **(C)** LFS-based RPA-CRISPR/Cas12a assays. No-crRNA means the reaction without crRNA. AHPND-crRNA means crRNA targeted to AHPND pathogen, EHP-crRNA and WSSV-crRNA targeted to EHP and WSSV pathogen, respectively. **(D)** Detection Ct value of qPCR in field samples. Field samples below the blue dashed line indicate the positive results. **(E)** Agreement between LFS-based RPA-CRISPR/Cas12a and qPCR assays for detecting AHPND in 60 field samples of shrimp. Error bars represent the standard deviation from three independent experiments.

We evaluated the specificity of the new RPA-CRISPR/Cas12a assay by examining cross-reactivity with two other pathogens that commonly affect penaeid shrimp, WSSV and EHP. As seen in Figures 4B,C, it showed positive results with significant fluorescence signal in Figure 4B and red line at the test band in Figure 4C when the reaction existed pathogen DNA with matched crRNA, both the fluorescence-based and LFS-based RPA-CRISPR/Cas12a assays did not cross-react with these two shrimp pathogens, demonstrating specificity for the *Vibrio* virulence genes target.

To evaluate the accuracy of the LFS-based RPA-CRISPR/Cas12a assay, we compared results with the real-time qPCR assay on 60 field samples of shrimp. qPCR detected 40 AHPND-positive and 20 AHPND-negative shrimp samples (Figure 4D). The LFS-based RPA-CRISPR/Cas12a assay detected 37 of the 40 AHPND-positive samples and all 20 of the AHPND-negative samples (Figure 4E). As seen in Table 1, the test results showed 92.5% positive predictive agreement (PPA) and 100% negative predictive agreement (NPA), respectively.

**Table 1:** Correlation of AHPND field-testing results for the LFS-based RPA-CRISPR/Cas12a assay and real-time qPCR.

		**Real-time qPCR**	
		**Positive**	**Negative**
LFS-based RPA-CRISPR/Cas12a	Positive	37	0
	Negative	3	20
		PPA: 92.5%	NPA: 100%

*PPA, positive predictive agreement; NPA, negative predictive agreement*.

## Discussion

While improvement of water quality and oxygen levels can reduce the concentration of AHPND-causing *Vibrio* (39), and farmers have used broad-spectrum antibiotics to inhibit gram-negative bacteria and parasites (40), these measures cannot prevent outbreaks of pathogenic bacteria. Furthermore, inappropriate use of broad-spectrum antibiotics may result in multidrug-resistant bacteria that exacerbate the risk of outbreaks. Therefore, timely and rapid detection of shrimp diseases at early stages of the infection is urgent to prevent outbreaks that have devastating economic consequences. Current AHPND tests include molecular assays (such as qPCR and nested PCR) and histological analysis [hematoxylin and eosin (H&E) staining]. qPCR can detect low levels of pathogens both qualitatively and quantitatively, and H&E staining is helpful for distinguishing between normal and necrotic tissues in photomicrographs. However, qPCR requires laboratory equipment and H&E staining is not useful for catching early-stage infections in shrimp samples, and both approaches are time consuming (41). Thus, there is a need for simpler, rapid tests that are suitable for use in a field setting and can detect low levels of pathogen prior to an AHPND outbreak.

To address this problem, we developed a new assay, the LFS-based RPA-CRISPR/Cas12a assay, by combining CRISPR/Cas12a with RPA and a rapid LFS-based readout that can be used for the field detection of shrimp AHPND (Figure 4A). The new assay includes three steps that take ~2 h: (1) DNA extraction from shrimp; (2) RPA amplification of target DNA; and (3) CRISPR/Cas12a reaction with LFS readout within 10 min. Compared with qPCR, RPA amplifies target DNA in 20–30 min at a constant temperature 37–42°C and thus does not require a thermocycler. In addition, the results of our assay can be visualized on LFS, making it a convenient and rapid alternative that can be translated to the field.

The advantage of our LFS-based RPA-CRISPR/Cas12a assay is apparent since it only requires three steps without using any complicated equipment. Additionally, more pathogens could be detected through the modification of crRNA sequences, which widen the detection scope. Furthermore, the crRNA used in the CRISPR/Cas12a reaction can trigger different cleavage activities of Cas12a, so careful selection of the crRNA is crucial for achieving robust cleavage activity to use in an assay (33).

To achieve high accuracy with our RPA-CRISPR/Cas12a assay, a few design factors needed to be taken into consideration. For instance, RPA is a crucial step that can pre-amplify low levels of target DNA present early in the course of an infection. Compared with PCR, the amplicon size of RPA may be constrained to under 1,000 bp, since its optimal amplification size is between 100 and 300 bp (42). The length of RPA primers is typically 30–35 bp. Thus, an amplicon size larger than 300 bp or primers smaller than 25 bp can reduce the efficiency of amplification. With these limitations, both target DNA sequence and primer length need to be taken into account to achieve the best performance. In addition, the test results derived from our CRISPR-based assay are more reliable when used in field assay of AHPND where the field conditions could be variable. For instance, RPA reaction works in the presence of three core enzymes. To avoid the RPA core enzymes and other reagents become inactive in transportation and longtime storage, the commercially purchased RPA components were lyophilized and all reagents used (antibodies on the test strips, Cas12a, crRNA and ssDNA reporters) were transported through cold-chain.

As for economic cost, compared with the qPCR—high-throughput at low cost (such as US$1-$5/test), our LFS-based RPA-CRISPR/Cas12a assay requires high expenditure of the reagents (RPA at the price of US$2/test and lateral flow strip at the price of US$3/test) and cold-chain transportation. However, instrument-dependent qPCR method can be expensive with equipment acquisition and maintenance. Besides, the laboratory method like qPCR cannot fulfill the instant detection of the field assay with drawbacks in the requirement of complicated equipment and technical expertise. Our LFS-based RPA-CRISPR/Cas12a assay provides the advantages of rapid, specific and portable which can fill this gap in first-line detection, thus, it may have important field applications for detecting AHPND in farmed shrimp.

In this study, We tested cross-reactivity with two other shrimp pathogens—WSSV and EHP—and showed high specificity of our RPA-CRISPR/Cas12a assay. Compared with qPCR, the LFS-based RPA-CRISPR/Cas12a assay also displayed high concordance with real-time qPCR results on field samples of shrimp, with 92.5% PPA and 100% NPA. The lower PPA between the LFS-based RPA-CRISPR/Cas12a assay and real-time qPCR may be due to the differences in the two amplification methods. Particularly, the amplification efficiency is highly affected by the primers used. The optimal length of RPA primers is 30–35 bp, but 20 bp for qPCR primers. The amplification of qPCR is driven by one key enzyme and thermocycling between 95 and 60°C, whereas RPA requires three enzymes and a constant temperature of 37°C (24). The quality of the field test samples may also have led to inconsistencies between test results. *Vibrio* DNA isolated from the field shrimp samples may have been fragmented that primer binding sites were lost, thus affecting the RPA reaction step. Sample preparation may need to be further optimized.

In summary, taking advantage of the high specificity of crRNA to target DNA, and the collateral cleavage activity of Cas12a, we developed a new AHPND molecular assay that combines CRISPR/Cas12a with RPA and readout by LFS. This new assay requires minimal equipment and involves a simple three-step process. Advantages relevant to field use include amplification at a constant temperature at 37–42°C and visualization of results by LFS. The LOD of the new RPA-CRISPR/Cas12a assay can reach the attomole level: 20 copies/μL for the fluorescence assay and 200 copies/μL for the lateral flow strip assay. This is comparable to the 200 copies/μL LOD of the current gold standard qPCR assays used to detect AHPND infection. Thus, our new LFS-based RPA-CRISPR/Cas12a assay is rapid, specific, and portable, and may be suitable for use for the field detection of shrimp AHPND.

## Data Availability Statement

The original contributions presented in the study are included in the article/Supplementary Material, further inquiries can be directed to the corresponding author/s.

## Author Contributions

QC, CL, and NL conceptualized and designed research. CL, ZF, MLin, BG, KC, and WL performed the experiments. NL, QW, MLi, and YY provided shrimp samples. QC and CL analyzed data and wrote the manuscript. All authors have read and approved the final manuscript.

## Funding

This work was supported by the Natural Science Foundation of Fujian Province, China (Grant No. 2021J01202).

## Conflict of Interest

The authors declare that the research was conducted in the absence of any commercial or financial relationships that could be construed as a potential conflict of interest.

## Publisher's Note

All claims expressed in this article are solely those of the authors and do not necessarily represent those of their affiliated organizations, or those of the publisher, the editors and the reviewers. Any product that may be evaluated in this article, or claim that may be made by its manufacturer, is not guaranteed or endorsed by the publisher.
