# Supplementary material for: CRISPR/Cas12a Based Rapid Molecular Detection of Acute Hepatopancreatic Necrosis Disease in Shrimp
Source: Front Vet Sci. 2022 Jan 25;8:819681. doi: 10.3389/fvets.2021.819681 (PMC8821903; doi:10.3389/fvets.2021.819681)
Supplement: Supplementary file 1 [file Data_Sheet_1.docx]

Supplementary Material


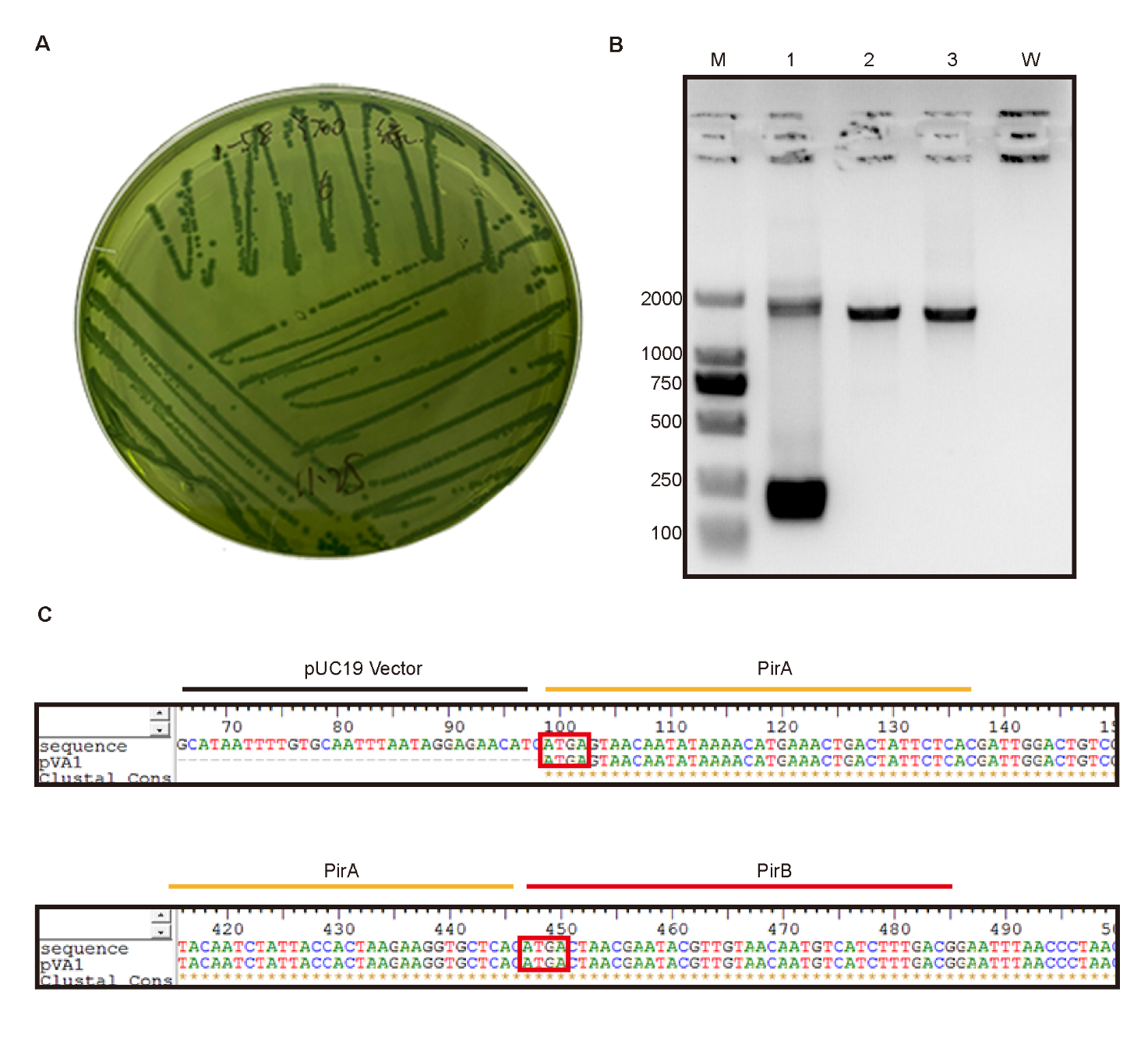


**Supplementary Figure 1.** Construction of pUC19-PirAB plasmid. **(A)** Isolation of *vibrios* with Thiosulfate Citrate Bile Salts Sucrose Agar (TCBS). **(B)** PCR products of *pir^VP^A* and *pir^VP^B* genes. **(C)** Sequencing of recombinant plasmid pUC19-PirAB.


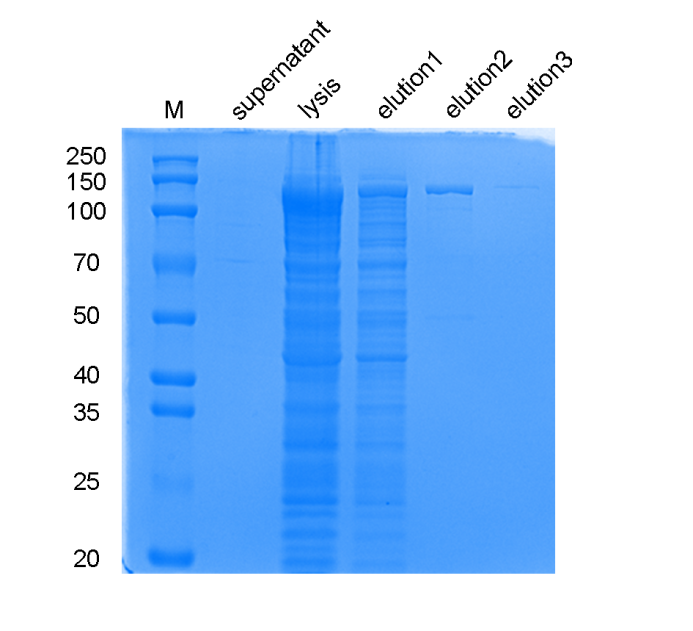


**Supplementary Figure 2.** Purification of LbCas12a protein. **(A)** SDS-PAGE gel of purified LbCas12a with His-tag.


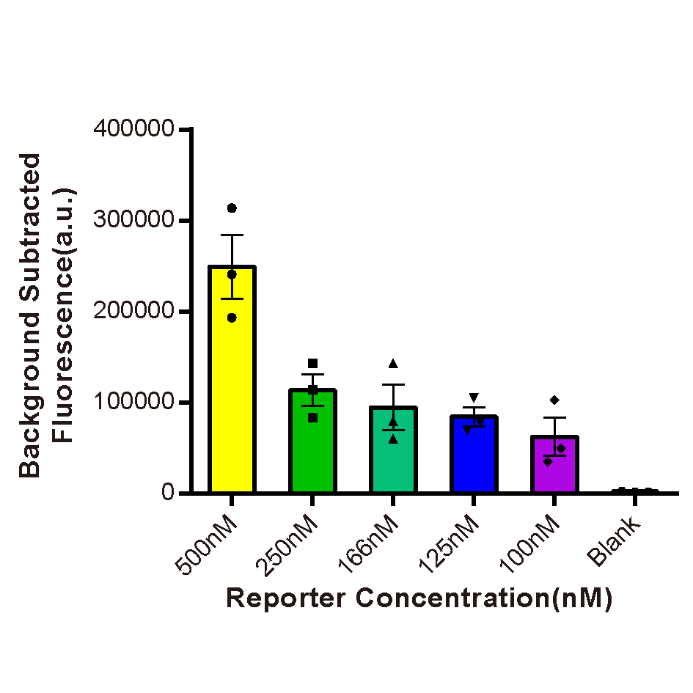


**Supplementary Figure 3.** Optimizing of FQ reporter. **(A)** Fluorescence assay in different concentration of FQ reporter. Error bars represent the standard deviation from three independent experiments.


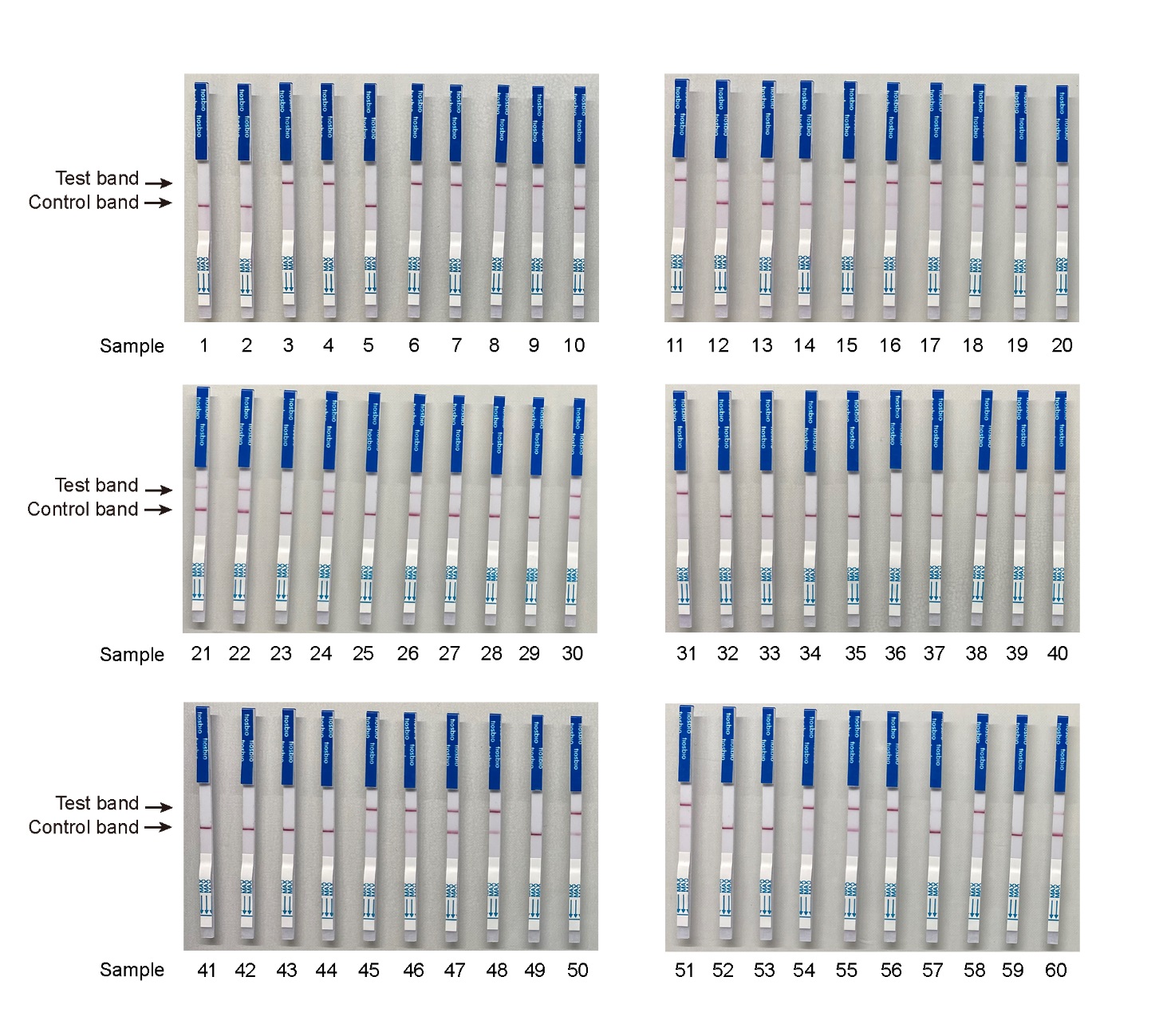


**Supplementary Figure 4.** Detection of AHPND in 60 field samples.

**Supplementary Table 1.** Primes used for PCR, RPA and qPCR

| Primer name | sequence（5’-3’） |
| --- | --- |
| PirAB-F1 | ACTTAAACGTGTTGCATA |
| PirAB-R1 | AGACATAGAATTAACGGTCTT |
| PirAB-qPCR-F | TTGGACTGTCGAACCAAACG |
| PirAB-qPCR-R | GCACCCCATTGGTATTGAATG |
| PirAB-RPA-F1 | TATGGCTGGCGGCTGGAAAGTGGCTAAATC |
| PirAB-RPA-R1 | CATGTGAGCACCTTCTTAGTGGTAATAGAT |
| PirAB-RPA-F2 | AACGGAGGCGTCACAGAAGTAGACAGCAAA |
| PirAB-RPA-R2 | TCGTTAGTCATGTGAGCACCTTCTTAGTGG |
| PirAB-RPA-F3 | TGCATAAGCTAACACGTAATATTGATAAGC |
| PirAB-RPA-R3 | GCAGGTGAATATGAAATTAAATTACTGTGAA |
| PirAB-RPA-F4 | CATTACAGAACTCGCTGCGTGGGCTGATAA |
| PirAB-RPA-R4 | AGGTATTTCATCTTCCGTACCTGTAGCAAT |
| PirAB-RPA-F5 | CAAACGGAGGCGTCACAGAAGTAGACAGCA |
| PirAB-RPA-R5 | TCGTTAGTCATGTGAGCACCTTCTTAGTGG |
| PirAB-RPA-F6 | GTGCATAAGCTAACACGTAATATTGATAAG |
| PirAB-RPA-R6 | GCAGGTGAATATGAAATTAAATTACTGTGAA |

**Supplementary Table 2.** Oligonucleotides used to form dsDNA templates for *in vitro* transcription

| Oligo | Sequence (5’ to 3’) |
| --- | --- |
| PirA-  crRNA1-F | GAAATTAATACGACTCACTATAGGGTAATTTCTACTAAGTGTAGATCAACGCCCTGATAATGCATT |
| PirA-  crRNA1-R | AATGCATTATCAGGGCGTTGATCTACACTTAGTAGAAATTACCCTATAGTGAGTCGTATTAATTTC |
| PirA-  crRNA2-F | GAAATTAATACGACTCACTATAGGGTAATTTCTACTAAGTGTAGATATCACGTTGTACCACATGTG |
| PirA-  crRNA2-R | CACATGTGGTACAACGTGATATCTACACTTAGTAGAAATTACCCTATAGTGAGTCGTATTAATTTC |
| PirB-  crRNA1-F | GAAATTAATACGACTCACTATAGGGTAATTTCTACTAAGTGTAGATAACATATGCTTATTTGGCAA |
| PirB-  crRNA1-R | TTGCCAAATAAGCATATGTTATCTACACTTAGTAGAAATTACCCTATAGTGAGTCGTATTAATTTC |
| PirB-  crRNA2-F | GAAATTAATACGACTCACTATAGGGTAATTTCTACTAAGTGTAGATAGATGATCGAACATTTGTTG |
| PirB-  crRNA2-R | CAACAAATGTTCGATCATCTATCTACACTTAGTAGAAATTACCCTATAGTGAGTCGTATTAATTTC |
| EHP-  crRNA-F | GAAATTAATACGACTCACTATAGGGTAATTTCTACTAAGTGTAGATGCCTCCGTTGGTCCAGGTGG |
| EHP-  crRNA-R | CCACCTGGACCAACGGAGGCATCTACACTTAGTAGAAATTACCCTATAGTGAGTCGTATTAATTTC |
| WSSV-  crRNA-F | GAAATTAATACGACTCACTATAGGGTAATTTCTACTAAGTGTAGATGCTATCTTCATAATCCATTG |
| WSSV-  crRNA-R | CAATGGATTATGAAGATAGCATCTACACTTAGTAGAAATTACCCTATAGTGAGTCGTATTAATTTC |
